# Involvement of signal peptidase I in *Streptococcus sanguinis* biofilm formation

**DOI:** 10.1099/mic.0.000516

**Published:** 2017-09-04

**Authors:** Jessica Aynapudi, Fadi El-Rami, Xiuchun Ge, Victoria Stone, Bin Zhu, Todd Kitten, Ping Xu

**Affiliations:** ^1^​ Philips Institute for Oral Health Research, Virginia Commonwealth University, Richmond, VA, USA; ^2^​ Department of Microbiology and Immunology, Virginia Commonwealth University, Richmond, VA, USA; ^‡^​ Present address: School of Dentistry, Virginia Commonwealth University, Richmond, VA, USA.

**Keywords:** biofilm, *Streptococcus sanguinis*, signal peptidase, RNA-seq, mass spectrometry

## Abstract

Biofilm accounts for 65–80 % of microbial infections in humans. Considerable evidence links biofilm formation by oral microbiota to oral disease and consequently systemic infections. *Streptococcus sanguinis*, a Gram-positive bacterium, is one of the most abundant species of the oral microbiota and it contributes to biofilm development in the oral cavity. Due to its altered biofilm formation, we investigated a biofilm mutant, Δ*SSA_0351*, that is deficient in type I signal peptidase (SPase) in this study. Although the growth curve of the Δ*SSA_0351* mutant showed no significant difference from that of the wild-type strain SK36, biofilm assays using both microtitre plate assay and confocal laser scanning microscopy (CLSM) confirmed a sharp reduction in biofilm formation in the mutant compared to the wild-type strain and the paralogous mutant ΔSSA_0849. Scanning electron microscopy (SEM) revealed remarkable differences in the cell surface morphologies and chain length of the Δ*SSA_0351* mutant compared with those of the wild-type strain. Transcriptomic and proteomic assays using RNA sequencing and mass spectrometry, respectively, were conducted on the Δ*SSA_0351* mutant to evaluate the functional impact of SPase on biofilm formation. Subsequently, bioinformatics analysis revealed a number of proteins that were differentially regulated in the Δ*SSA_0351* mutant, narrowing down the list of SPase substrates involved in biofilm formation to lactate dehydrogenase (SSA_1221) and a short-chain dehydrogenase (SSA_0291). With further experimentation, this list defined the link between *SSA_0351*-encoded SPase, cell wall biosynthesis and biofilm formation.

## Introduction

A biofilm is an accumulation of uni- or polymicrobial species embedded in a protective extracellular polymeric matrix that adheres to biotic or abiotic surfaces [1] and forms a nutrient-sufficient ecosystem as a sessile microbial community. According to estimates by the US Centers for Disease Control and Prevention and the National Institutes of Health, biofilms account for 65–80 % of microbial infections in human beings [1, 2]. Infections that are biofilm-based have been discovered in almost all tissues of the human body [1]. Many studies have shown considerable evidence linking biofilm formation in the oral cavity to oral disease and consequently systemic infections. These systemic conditions include cardiovascular disease, diabetes mellitus, preterm births or low birth weights, rheumatoid arthritis and infective endocarditis [1, 3]. Biofilms possess a number of properties that make them resistant to treatment; therefore, eradication of biofilm-associated infections is difficult [1]. Cells in a biofilm exhibit increased tolerance to antibiotics as the biofilm matrix decreases the antibiotic diffusion rates to the physiologically dormant persister cells that are inherently resistant to antibiotics. Moreover, biofilm establishment provides a survival advantage as a defence system against host immune defences such as macrophages [4–6].

The highly developed oral microbiome is a diverse environment that is largely influenced by oral streptococci [7, 8]. *Streptococcus sanguinis*, a Gram-positive bacterium, is a known pioneer colonizer of oral surfaces and one of the most abundant species in oral biofilm [9, 10]. After initially colonizing the tooth surface and forming dental plaque, an oral biofilm, *S. sanguinis* and other endogenous streptococci serve as a tether for the attachment of other micro-organisms to a salivary glycoprotein-coated surface [8, 11]. One of these micro-organisms is *Streptococcus mutans*, whose overgrowth is often associated with the development of dental caries [12, 13]. We presume that the ability of *S. sanguinis* to interfere with the colonization of *S. mutans* on teeth may be beneficial for oral health [11, 14].

Oral biofilm formation is a demanding task and requires *S. sanguinis* to harness a network of genes, one of which is signal peptidase I (SPase), a serine membrane-bound endoprotease that cleaves off a signal peptide from the majority of secreted proteins. Almost one-third of all synthesized bacterial preproteins, destined to function in the cell membrane or extracellular milieu, need to be translocated through the cytoplasmic membrane, mostly via the general secretion pathway (Sec pathway), or in some cases via the twin arginine translocation (tat) pathway [15]. The signal peptide sequence, a ‘zipcode’ that guides the preproteins to the Sec or Tat membrane-embedded pore (translocon), is cleaved by SPase during or shortly after translocation, releasing the translocated protein from the membrane and allowing its folding into a mature protein. SPases represent an attractive target for drug development for many reasons: they are ubiquitous [16]; often essential [17]; accessible to drugs as their active sites are exposed to the extracellular region [18, 19]. Bacterial species such as *E. coli* have only one essential SPase enzyme, while most Gram-positive bacteria have multiple enzymes [15, 20–22]. *S. sanguinis* possesses two homologous SPases encoded by *SSA_0351* and *SSA_0849* that target a set of 168 potential substrates, as predicted by LipoP software. We observed that SSA_0351 is essential for biofilm formation in *S. sanguinis*. We also identified transcriptional changes and changes in protein abundance that may explain the biofilm defect. Identifying which SPase in *S. sanguinis* contributes to biofilm formation and delineating the underlying mechanism will enhance our understanding of bacterial biofilm formation and pave the way to effectively prevent and treat many oral infections, as well as many systemic infections that originate in the oral cavity.

In conclusion, through elucidating the role of *SSA_0351* in biofilm formation in *S. sanguinis*, we promote its candidacy as a promising drug target and help characterize a bacterial model for further studying the genetic matrix that contributes to biofilm structure and function.

## Methods

### Bacterial strains, media and growth conditions

*S. sanguinis* SK36 and all mutants (single gene knockouts) were routinely grown in brain heart infusion (BHI) broth (BD, San Jose, CA, USA) under microaerobic conditions (7.2 % H_2_, 7.2 % CO_2_, 79.6 % N_2_ and 6 % O_2_) at 37 °C as previously described [10]. For the construction of single-gene knockouts, the open reading frame (ORF) of a single gene in *S. sanguinis* SK36 was replaced by a promoterless kanamycin cassette (*aphA-3*) as described previously [23]. For example, the *SSA_0351* mutant was constructed as Kan^r^; Δ*SSA_0351 *:: aphA-3, and will be referred to as Δ*SSA_0351*. For mutant culture, the medium was supplemented with kanamycin (Sigma-Aldrich, CA, USA) (500 µg ml^−1^). Biofilm medium supplemented with 1 % (w/v) sucrose (BM) was used for biofilm formation [10].

For the complementation of Δ*SSA_0351*, three DNA fragments were independently amplified using the primer sets F1-0351 (CCGTCTCTGTCAAGGTAGCC)/R1-0351 (TGTAATCACTCCTTCTCACTATTTATTAATAGGTCTTAAAAGGCAGCAGT), F2-erm (TAAATAGTGAGAA GGAGTGATTACATGAACAA)/R2 erm (TTATTTCCTCC CGTTA AATAATAG) and F3-0351 (CTATTATTTAA CGGGAGGAAATAAGGCATACCGAACTC AGCTGA AC)/R3-0351 (TGGGCATAGACTGCGATG) for the 1 kb sequence upstream plus the coding sequence of SSA_0351, the erythromycin resistance cassette (pVA838) and the 1 kb sequence downstream of SSA_0351, respectively. The final recombinant PCR product containing these three fragments was produced by overlapping PCR and then introduced into Δ*SSA_0351* to replace the kanamycin resistance cassette with the SSA_0351 ORF and the erythromycin resistance cassette. An erythromycin-resistant and kanamycin-sensitive transformant Δ*SSA_0351_*C (Erm^r^; SSA_0351*^+ ^*:: *pSerm*) was selected and confirmed by PCR analysis.

### Data mining from databases

We searched for proteins homologous to SSA_0351 in the *S. sanguinis* SK36 genome using the blastp algorithm. LipoP 1.0 software (http://www.cbs.dtu.dk/services/LipoP/) was used to predict the substrates of signal peptidases I and II of all *S. sanguinis* SK36 proteins [24]. Although this program has been trained on Gram-negative bacterial protein sequences, its performance on Gram-positive bacterial protein sequences was acceptable. Multiple protein sequence alignments of *S. sanguinis* SPases were conducted using MultAlin version 5.4.1 [25] and a phylogenic tree based on hierarchical clustering was constructed showing the evolutionary relatedness of various bacterial SPases. The evolutionary distances between SPases have been calibrated to 10 PAM, where 1 per cent accepted mutation (PAM) is the time in which 1 amino acid substitution event per 100 sites is expected to have happened. In other words, a PAM unit is the ‘evolutionary unit’ that will change 1 % of the amino acids within a protein sequence [26].

### Biofilm assay

The biofilm formation of *S. sanguinis* SK36 and mutants was examined in 12-well microtitre plates (Greiner Bio-One, Monroe, NC, USA) using the O’Toole method to observe bacterial adherence to an abiotic surface [27]. Bacterial strains were cultured overnight, diluted 100-fold in BM-loaded microtitre plates and incubated 16 h at 37 °C under anaerobic conditions (10 % CO_2_, 10 % H_2_ and 80 % N_2_ with a catalyst) for biofilm formation. Bacterial growth was measured at 600 nm absorbance using a Synergy H1 hybrid reactor (BioTek, VT, USA) microplate reader and the plate wells were gently washed with deionized water (dH_2_O) to remove the remaining planktonic cells, and stained with 50 µl of 0.4 % (w/v) crystal violet solution (Fisher Scientific, Pittsburgh, PA, USA) for 15 min at room temperature. After being washed three times with dH_2_O, the biofilm stain was solubilized in 200 µl of 33 % (v/v) acetic acid for 30 min. Finally, 100 µl from each well was transferred to a new plate well for absorbance to be measured at 600 nm. Each strain was tested in eight replicates.

### Confocal laser scanning microscopy (CLSM) and image analysis

For confocal laser scanning microscopy, 6 wells in a 12-well microtitre plate were filled with 1 ml of BM and inoculated with 10 µl of overnight-grown bacterial culture. The first column contained *S. sanguinis* SK36 and the second contained a mutant for three repeats each. After overnight incubation, biofilms were rinsed once with 1 ml phosphate buffered saline (PBS) to remove the unattached bacteria. For 15 min, biofilms were labelled with 1.5 µM SYTO9 (Life Technologies, Grand Island, NY, USA), a green fluorescent dye that detects live cells. Afterwards, the wells were rinsed with 1 ml PBS to remove the remaining dye. Biofilms were viewed through a 10× dry lens with a Zeiss LSM 710 confocal laser scanning microscope (VCU core facilities). Green fluorescence was imaged and an image stack of a randomly chosen spot was collected for each sample using a laser wavelength of 488 nm and emission wavelengths of 495–525 nm. A series of green fluorescent *x–y* sections in the *z* plane of the biofilm were scanned and obtained. Images were analysed with Image J v 1.47 (National Institutes of Health). The biofilm thickness and roughness coefficient parameters of the biofilms were measured using MATLAB.

### Scanning electron microscopy (SEM) analysis of *S. sanguinis* SK36 and mutants

Overnight cultures of Δ*SSA_0351* and *S. sanguinis* SK36 were diluted 1 : 100 in BHI and grown to late log phase. Bacterial samples were deposited onto a 0.1 µm disposable Millipore filter to remove medium. Samples were fixed using 2 % glutaraldehyde in 0.1 M sodium cacodylate buffer (pH 7.4) for 30 min, followed by 1 % osmium tetroxide in 0.1 M sodium cacodylate buffer (pH 7.4). The samples embedded in the filters were then dehydrated in ethanol followed by PBS and allowed to air dry. The filters were sectioned and mounted onto stubs and coated with gold for 3 min (EMS– 550 Automated Sputter Coater, Electron Microscopy Sciences, Hatfield, PA, USA). Micrographs were taken at 10 000 and 20 000× total magnification using a Zeiss EVO 50 XVP scanning electron microscope (Carl Zeiss, Peabody, MA, USA).

### Examination of Δ*SSA_0351* growth *in vitro*

Overnight cultures of Δ*SSA_0351* and *S. sanguinis* SK36 were diluted 1 : 100-fold in BHI and grown for 4 h in microaerobic conditions before beibg diluted 20-fold into microplate wells containing fresh BHI. Each sample was tested in triplicate. Growth rates were determined by measuring the OD_600_ using a Synergy H1 hybrid reactor microplate reader (BioTek, VT, USA) every 10 min under aerobic conditions for 14 h. The experiment was performed in triplicate.

### Auto-aggregation assay

The auto-aggregation ability of *S. sanguinis* mutants with respect to wild-type was measured using the auto-aggregation assay as described by Luo *et al*. [28]. Bacterial samples were grown overnight in BHI medium under microaerophilic conditions. After 24 h, the cultures were agitated vigorously and the turbidity of each sample was measured (A0) at 600 nm using a UV/visible spectrometer (Biomate 3S, Thermo Scientific, USA). After 8 h at room temperature, the absorbance (A8) of the culture was measured again. Auto-aggregation ability was expressed as auto-aggregation percentage (Ag %) and calculated using Ag %=[(A0−A8)/A0]×100.

### Transcriptome analysis by RNA-seq

For RNA-seq, three replicates of *S. sanguinis* SK36 and Δ*SSA_0351* cultures were grown overnight in BHI broth at 37 °C in microaerophilic conditions. The next day, cells were diluted 1 : 100 into 5 ml BHI broth and grown at 37 °C for 4.5–5 h until OD_600_ of 0.6 was attained, after which 10 ml of RNAprotect bacteria reagent (cat. #76506, Qiagen, CA, USA) was added to each bacterial culture. Cells were incubated for 5 min at room temperature and centrifuged, with the pellet then being stored at −80 °C. Cells were lysed using RNeasy mini kit (cat. #74106, Qiagen, CA, USA) as recommended by the manufacturer and by bead milling conducted using 2 ml lysing matrix B beads in the FastPrep 24 for 45 s at level 6. All samples were treated with the DNase I RNase-free DNase set (cat. #79254, Qiagen, CA, USA) to deplete DNA. Total RNA concentrations were measured using a NanoDrop 2000 UV/Vis spectrophotometer (Thermo Fisher, DE, USA) with cutoff values for the absorbance ratios of 260/280 and 260/230 of 2.0 and 2–2.2, respectively. For the depletion of ribosomal RNA, all samples were treated with the Illumina Ribo-zero Magnetic Kit for Bacteria (cat. #MRZB12424, Roche, USA) and the rRNA-depleted samples were purified using the Qiagen RNeasy MinElute Cleanup kit (cat. #74204, Qiagen, CA, USA). RNA concentrations were measured in rRNA-depleted samples using the NanoDrop 2000 UV/Vis spectrophotometer with cutoff values for RNA concentration of 10 ng µl^−1^. Actinomycin D (cat. #A1410-2MG, Sigma-Aldrich, MO, USA) was used for RNA fragmentation and RNA libraries were prepared with NEBNext Ultra Directional RNA Library Prep Kit NEB (cat.# E7420L, New England Biolabs, MA, USA) and NEBNext Multiplex Oligos for Illumina Index Primers sets 1 and 2 (cat. #E7335L and E7500L, respectively, New England Biolabs, MA, USA). The final cDNA products were purified with AMPure XP beads (cat. #A63880, Beckman Coulter, CA, USA) and band sizes were checked by gel electrophoresis. The quality of the constructed cDNA library was determined using Agilent Bioanalyzer-High Sensitivity DNA Chip and Ribosome Integrity Numbers (RIN) were determined for all samples with a cutoff value of 10. Sequencing was carried out on an Illumina MiSeq platform using reagent kit v 2, with coverage of ~15 M reads each. The reads obtained from sequencing were aligned against the *S. sanguinis* SK36 genome using BasePairtech software and counts of transcripts along with statistical calculations were provided.

### Gene expression data

The RNA-seq data were deposited in the Gene Expression Omnibus (GEO) database (http://www.ncbi.nlm.nih.gov/geo/) under the accession number GSE81238.

### Proteomic analysis

Protein samples were prepared from bacterial lysates as follows. Overnight cultures of *ΔSSA_0351* and *S. sanguinis* SK36 were diluted 100-fold into 50 ml BHI for 5 h of growth under microaerobic conditions. Bacterial cells were centrifuged for 10 min at 4 000 r.p.m. using a Sorvall Legend RT centrifuge (MN, USA) at 4 °C, washed twice with cold PBS and mixed with lysis buffer (50 mM Tris-HCl, 150 mM NaCl, 1 % SDS, 1 mM dithiothreitol) supplemented with protease inhibitor cocktail (Sigma P8430). After 30 min on ice, the resuspended pellets were transferred into 2 ml Lysing Matrix B tubes. Cells were disrupted in a FastPrep 24 for 40 s at level 4.5 twice, and samples were cooled by ice for 5 min in-between. Lysates were centrifuged for 15 min at 13 000 r.p.m. at 4 °C and the supernatant was transferred into a new tube. Soluble protein was quantitated using Pierce BCA protein assay kit (cat. #23227, IL, USA) at 562 nm absorbance after 30 min incubation at 37 °C with BSA as a standard.

Aliquots were removed from each sample and subjected to acetone precipitation. To aid in digestion, the samples were resuspended in ammonium bicarbonate and Rapigest. The samples were reduced with 10 mM dithiothreitol in 0.1 M ammonium bicarbonate at room temperature for 30 min. Then they were alkylated with 50 mM iodoacetamide in 0.1 M ammonium bicarbonate at room temperature for 30 min. The samples were digested with 1 µg trypsin twice overnight and then quenched with 5 % (v : v) glacial acetic acid.

The injection volume was adjusted to achieve 100 ng protein on-column per injection. Each sample was run in triplicate. The samples were analysed by a Waters Synapt G2Si mass spectrometer system with a nanospray ion source interfaced to a Waters M-Class C18 reversed-phase capillary column. The peptides were injected onto the trap and analytical columns, and the peptides were eluted from the column by an acetonitrile/0.1 % formic acid gradient at a flow rate of 0.4 µl min^−1^ over 60 min. The digests were analysed using the double-play capability of the instrument, acquiring full scan mass spectra at low collision energy to determine peptide molecular weights and product ion spectra at high collision energy to determine the amino acid sequence. The ion mobility mode was used to produce a third dimension of separation, maximizing the number of peptide identifications.

Progenesis QI software was used to perform label-free relative quantification, aligning chromatographic peaks, normalizing across samples and then reporting out the proteins that had changed in abundance. The data were analysed by database searching using the PLGS search algorithm against Uniprot’s *S. sanguinis* database.

### Statistical analysis

For quantitation of biofilm formation using the biofilm assay, the results from microtitre staining were analysed statistically by ANOVA and Dunnett's test. For the CLSM analysis, the image stacks of biofilms grown by each mutant were compared to that of *S. sanguinis* SK36. The significance was set for Student’s *t*-test results with *P*-value <0.05.

## Results

### Database mining to investigate functional cues about SPase SSA_0351

SPases are important contributors to cellular proteostasis, impacting on all aspects of bacterial behaviour, including biofilm formation, which is proposed to be the predominant lifestyle of bacteria in diverse environments. In this study, we proposed a model to characterize the contribution of *SSA_0351*-encoded SPase to the formation of biofilms on solid surfaces.

**Fig. 1.**
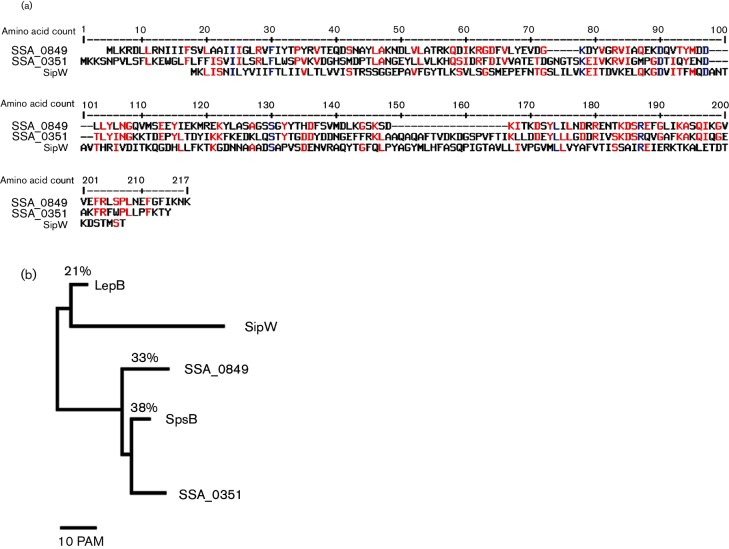
Characteristics of *SSA_0351*-encoded SPase as extracted from various databases. (a) Multiple protein sequence alignments of *S. sanguinis* SPases, SSA_0351 and SSA_0849, and *B. subtilis* SipW were conducted using MultAlin. Blue: identity across three sequences. Red: identity between two sequences. (b) Phylogenic tree showing the evolutionary relatedness of bacterial SPases (LepB from *Escherichia coli* str. K-12 substr. MG1655, SpsB from *Staphylococcus aureus* subsp. aureus NCTC 8325, SipW from *Bacillus subtilis*) based on protein sequence alignment with hierarchical clustering. The identity percentages of the protein sequence alignments for every SPase with respect to SSA_0351 are shown, except for SpiW, which was too low. The evolutionary distances between SPases have been calibrated to 10 PAM, where 1 per cent accepted mutation (PAM) is the time in which 1 substitution event per 100 sites is expected to have happened.

Using data mining, we defined all putative SPase-coding genes in *S. sanguinis* SK36, *SSA_0351* and *SSA_0849*. We compared the protein sequences of both SPases in *S. sanguinis* using MultAlin software (Fig. 1a) and 33 % protein sequence identity was shared between the two SPases. Comparing other functionally investigated bacterial SPases (LepB from *Escherichia coli* str. K-12 substr. MG1655, SpsB from *Staphylococcus aureus* subsp. aureus NCTC 8325 and SipW from *Bacillus subtilis* subsp. subtilis str. 168) to SSA_0351, we constructed a phylogenic tree based on hierarchical clustering to show the evolutionary relatedness of these SPases (Fig. 1b). Interestingly, SpsB, the major SPase in *S. aureus,* showed closer evolutionary distance to *SSA_0351*-encoded SPase than paralogous protein SSA_0849. This finding provided a potent clue about the putative function of SSA_0351, a signal peptidase, that has not been experimentally verified yet.

Using the LipoP 1.0 algorithm, which predicts substrates for signal peptidase I and II, we identified potential targets for SPase among *S. sanguinis* proteins (Table S1, available with the online Supplementary Material).

### Comparative analysis of biofilm formation by SPase mutants

**Fig. 2.**
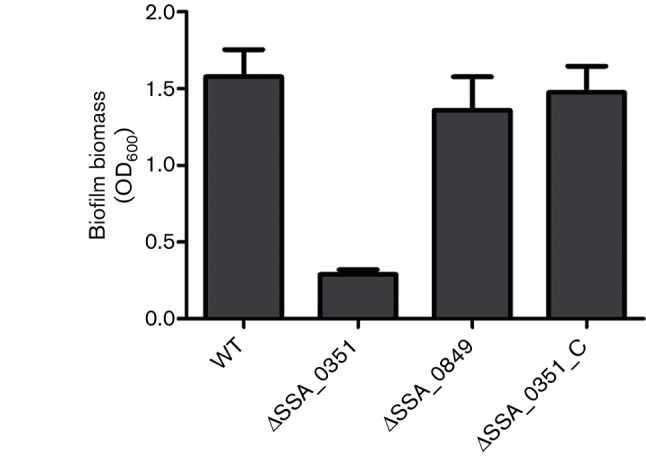
SPase-related mutants show different patterns of biofilm formation. Bacterial samples of eight replicates each were cultured anaerobically for 24 h in BM with 1 % sucrose. After crystal violet staining, biofilm formation was quantified at OD_600_ and the results were compared using ANOVA and the multiple comparison method (Dunnett's test). ** indicates significance with *P*-value <0.01.

The GenBank annotations of the *S. sanguinis* genome reveal two SPase-encoding paralogous genes in the *S. sanguinis* SK36 strain. To detect which paralogue (*SSA_0351* or *SSA_0849*) impacts on biofilm formation, we compared biofilms formed by two SPase mutants, namely *ΔSSA_0351* and *ΔSSA_0849*, with the wild-type, first using the biofilm assay to identify statistically significant difference in biofilm formation (Fig. 2). We detected a statistically significant (*P* value<0.05) decrease in biofilms formed by Δ*SSA_0351*, while *ΔSSA_0849* was not significantly different from the wild-type SK36. To rule out the polar effect due to deletion of SSA_0351, we complemented the Δ*SSA_0351* mutant. Biofilm formation by Δ*SSA_0351_*C was comparable to that of the wild-type (Fig. 2). In addition, the planktonic cells of the wild-type and mutants showed comparable growth (Table S2).

**Fig. 3.**
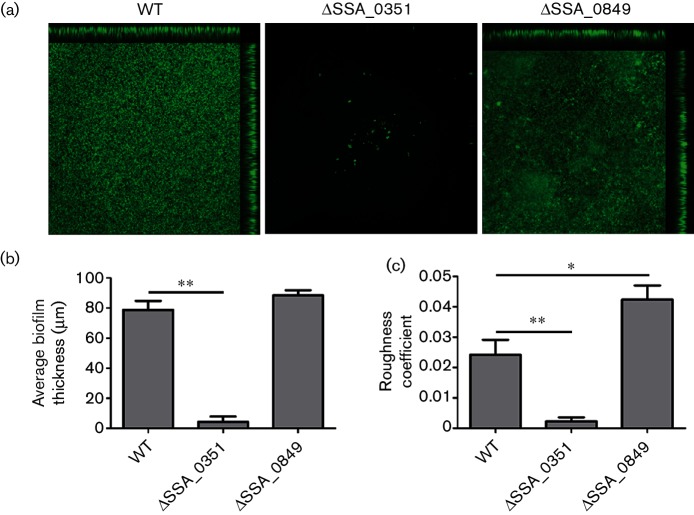
SPase-related mutants form different biofilm biomasses. Biofilm formation comparison of the SPase mutants and SK36 by confocal laser scanning microscopy (CLSM). CLSM imaging was conducted in triplicate and the images were analysed with Image J. (a) Representative images from wild-type and two SPase-related mutants, *ΔSSA_0351* and *ΔSSA_0849* are shown. (b) Biofilm thickness and (c) roughness coefficient parameters were measured using MATLAB. * indicates significance with *P*-value <0.05. **indicates significance with *P*-value <0.01.

To confirm our biofilm assay results, we compared the biofilms of mutants using CLSM imaging. The *z*-stacks were created by stacking successive slices, which were then processed into a 3D image using ImageJ software (Fig. 3a). In concordance with the biofilm assay findings, *ΔSSA_0351* demonstrated significantly reduced biofilm formation upon CLSM imaging in comparison to the wild-type strain, unlike *ΔSSA_0849*. Quantitative analysis of the biofilms showed that *ΔSSA_0351* did not form biofilms with measurable thickness, unlike *ΔSSA_0849*. The average thickness of the *ΔSSA_0849* biofilm was comparable to that of the wild-type (Fig. 3b). Moreover, the roughness coefficient of the *ΔSSA_0849* biofilm was almost double that of the wild-type, reflecting a smoother surface (Fig. 3c). Based on these findings, we further focused on *ΔSSA_0351* for its role in biofilm formation.

### Examination of Δ*SSA_0351* growth *in vitro*

**Fig. 4.**
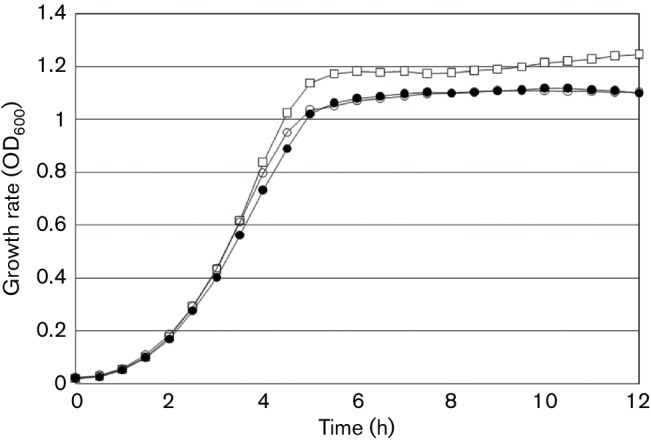
Growth curves of wild-type (WT), Δ*SSA_0849* and Δ*SSA_0351*. The growth rates of the wild-type strain and mutants were compared at different time intervals (hours). The growth curve assay was performed in triplicate and the average of the three experiments is shown here. Legends: empty square, WT; empty circle, *ΔSSA_0351*; filled circle, *ΔSSA_0849*.

To rule out the possibility that the reduction in the Δ*SSA_0351* growth rate was behind the reduction in biofilm formation, we compared the growth patterns of Δ*SSA_0351* and wild-type in triplicate over a period of 12 h using a plate reader (Fig. 4). Three independent trials showed that the growth rates of the wild-type, Δ*SSA_0849* and Δ*SSA_0351* strains were not significantly different, highlighting the fact that the reduced biofilm formation of the mutant cannot be attributed to slower mutant growth in comparison to the wild-type.

### SEM analysis of Δ*SSA_0351*

**Fig. 5.**
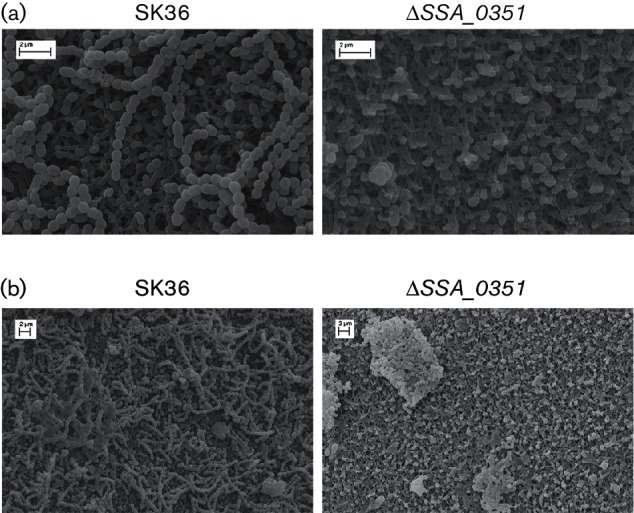
Scanning electron microscopy (SEM) analysis of the wild-type and Δ*SSA_0351* strain showed distorted cell morphology and the absence of cellular chains in the SPase-mutant Δ*SSA_0351*. Biofilms formed by the wild-type strain and Δ*SSA_0351* were scanned under (a) 20 000× magnification and (b) 10 000× magnification.

To further examine the changes to the mutant biofilm morphology, SEM analysis was employed. Representative SEM micrographs of wild-type SK36 and Δ*SSA_0351* strains are shown in Fig. 5. The micrographs of the wild-type strain demonstrated characteristic long chains of streptococcal cells, with spherical smooth cell walls of similar sizes. The cells and chains were uniformly distributed over the surfaces. The chains aggregated together to form a dense meshwork that masked the background.

The micrographs of Δ*SSA_0351* showed isolated bacterial cells that failed to form chain structures. The cell sizes were irregular and the surfaces of the cell walls appeared rough and shrunken. The cells were irregularly distributed, forming clusters that were not bound together.

In conclusion, the 3D cellular architecture of Δ*SSA_0351* was starkly different from that of the wild-type, and this may be attributed to the irregular sizes and surfaces of mutant cells.

### Transcriptomic analysis of Δ*SSA_0351*

We compared the transcriptomic profile of Δ*SSA_0351* with that of the wild-type using RNA-seq (Table 1). Based on the findings of Terra *et al*. [29], we suspected a role for *SSA_351*-encoding SPase on a transcription factor (or factors), which in turn impacts on the genes involved in biofilm formation. Categorizing genes with at least 1.5-fold changes and *P*-value <0.001, we identified the genes (*SSA_2141* and*SSA_2142*) involved in arginine metabolism, as shown by the KEGG database (http://www.genome.jp/kegg/), to be transcriptionally down-regulated. As for the up-regulated mRNA transcripts, it was shown that the genes involved in amino acid ABC transport (*SSA_1567-8-9*), an SOS response protein-encoding gene (*SSA_0621*) and a protease-encoding gene (*SSA_2096*) were significantly up-regulated. Although the SSA_1567 protein is a component of amino acid ABC transporter and a substrate of SPase, the transcriptional up-regulation in Δ*SSA_0351* warrants further investigation.

**Table 1.** List of transcripts that are differentially regulated in Δ*SSA_*0351

Gene symbol	Fold change*	*P*-value†	Gene description	SPase substrate‡
*SSA_0350*	0.022	0	Helicase	No
*SSA_1224*	0.594	2.72E-09	Hypothetical protein	No
*SSA_0670*	0.595	1.42E-19	Hypothetical protein	No
*SSA_1032*	0.638	4.68E-12	30S ribosomal protein S20	No
*SSA_2142*	0.649	1.09E-18	Argininosuccinate synthase	No
*SSA_2141*	0.657	3.25E-11	Argininosuccinate lyase	No
*SSA_1098*	0.658	1.32E-08	Formate/nitrate transporter	No
*SSA_2206*	1.502	7.86E-06	Hypothetical protein	No
*SSA_2149*	1.504	2.94E-06	Hypothetical protein	No
*SSA_0620*	1.505	6.99E-08	Hypothetical protein	No
*SSA_2207*	1.509	5.21E-06	Hypothetical protein	No
*SSA_0819*	1.516	6.43E-10	Hypothetical protein	No
*SSA_0618*	1.565	6.84E-07	Hypothetical protein	No
*SSA_1567*	1.571	8.22E-10	Polar amino acid ABC transporter amino acid-binding protein	Yes
*SSA_0621*	1.592	6.52E-14	SOS responce UmuC protein	No
*SSA_0614*	1.595	2.34E-09	Transporter	No
*SSA_1569*	1.603	9.98E-11	Arginine/histidine ABC transporter permease	No
*SSA_1568*	1.615	2.63E-14	Arginine/histidine ABC transporter permease	No
*SSA_2096*	2.016	8.18E-13	ATP-dependent protease, ATP- binding subunit	No

*Values are Δ*SSA*_0351:SK36. Cutoff for fold change is 1.5-fold decrease or increase.

†Cutoff for *P*-value was <0.001.

‡As predicted by LipoP 1.0 software.

In conclusion, the transcriptomic analysis pointed to the transcriptional down-regulation of two genes involved in arginine biosynthesis.

### Proteomic analysis of Δ*SSA_0351*

SPase is indispensable for the cleavage of signal peptide from its targets, which in turn starts the folding process of cleaved peptides and releases them to their final destinations. We hypothesized that in the absence of an SPase encoded by *SSA_0351*, its respective targets would be mostly misfolded and consequently trapped in translocons or degraded, or would accumulate inside the cell cytoplasm. To assess this hypothesis, we extracted the intracellular proteome of the Δ*SSA_0351* mutant to identify differentially abundant proteins in the mutant using mass spectrometry peptide sequencing. Progenesis software detected 61 protein changes (Table 2).

**Table 2.** Differentially expressed proteins in *ΔSSA_0351* using mass spectrometry

Protein symbol	Fold change	Score*	Protein description	SPase substrate†
SSA_1748	0.00000234	5.61	Probable manganese-dependent inorganic pyrophosphatase	No
SSA_1938	0.05	5.43	Enoyl-[acyl carrier protein] reductase II	No
SSA_1498	0.12	12.34	50S ribosomal protein L20	No
SSA_2107	0.17	10.71	Glutamine–fructose-6-phosphate aminotransferase	No
SSA_0570	0.23	5.46	Glutamyl-tRNA(Gln) amidotransferase subunit A	No
SSA_0291	0.31	5.53	Short-chain dehydrogenase	Yes
SSA_1012	0.42	5.99	Phosphoenolpyruvate–protein phosphotransferase	No
SSA_2342	0.57	5.12	VTC domain protein	No
SSA_2359	0.63	10.02	tRNA uridine 5-carboxymethylaminomethyl modification enzyme MnmG	No
SSA_1528	0.63	5.16	Phosphoglycerate mutase	No
SSA_0371	0.64	10.9	Glutamate dehydrogenase	No
SSA_2183	0.76	28.3	Glucose-6-phosphate isomerase	No
SSA_0126	0.8	26.5	50S ribosomal protein L15	No
SSA_2126	0.97	5.44	TatD family deoxyribonuclease	No
SSA_2069	1.06	5.79	Proline–tRNA ligase	No
SSA_0117	1.14	58.35	50S ribosomal protein L14	No
SSA_1998	1.14	32.74	Trigger factor	No
SSA_0357	1.2	12.02	Thioredoxin	No
SSA_0226	1.34	56.08	60 kDa chaperonin	No
SSA_2075	1.53	5.49	Transketolase	No
SSA_0848	1.59	149.33	Pyruvate kinase	No
SSA_2007	2.06	39.14	Chaperone protein DnaK	No
SSA_1968	2.21	11.34	Ketol-acid reductoisomerase	No
SSA_1836	2.25	11.05	Competence protein FA	No
SSA_0110	2.26	130.27	50S ribosomal protein L2	No
SSA_0847	2.34	27.94	ATP-dependent 6-phosphofructokinase	No
SSA_1948	2.35	11.69	Oligopeptide-binding protein SarA	No
SSA_0768	2.35	16.49	Ribonucleoside-diphosphate reductase	No
SSA_0132	2.39	27.09	DNA-directed RNA polymerase subunit alpha	No
SSA_2202	2.43	40.83	Elongation factor Ts	No
SSA_0440	2.54	32.15	30S ribosomal protein S18	No
SSA_1520	2.56	195.25	Elongation factor Tu	No
SSA_0683	2.57	91.7	DNA-binding protein HU	No
SSA_0141	2.72	5.57	Copper chaperone	No
SSA_1032	2.85	7.03	30S ribosomal protein S20	No
SSA_0098	3.04	5.18	HAD superfamily hydrolase	No
SSA_0130	3.2	30.68	30S ribosomal protein S13	No
SSA_0112	3.3	24.53	50S ribosomal protein L22	No
SSA_1204	3.4	23.43	Phosphoglucomutase	No
SSA_1925	3.5	11.65	Serine–tRNA ligase	No
SSA_1950	3.81	11.79	Oligopeptide ABC superfamily ATP binding cassette transporter, binding protein	No
SSA_0774	4.02	224.38	Glyceraldehyde-3-phosphate dehydrogenase	No
SSA_1992	4.25	171.16	Fructose-bisphosphate aldolase	No
SSA_0886	4.58	168.8	Enolase	No
SSA_0302	4.74	89.05	Phosphoglycerate kinase	No
SSA_2033	4.75	37.55	30S ribosomal protein S9	No
SSA_1619	4.79	5.67	Ribosome-recycling factor	No
SSA_1839	4.83	57.73	Cysteine synthase	No
SSA_1221	4.98	22.09	l-lactate dehydrogenase	Yes
SSA_0176	5.07	21.1	DNA-directed RNA polymerase subunit beta	No
SSA_1062	5.75	11.69	50S ribosomal protein L27	No
SSA_0688	5.77	32.78	2,3-bisphosphoglycerate-dep phosphoglycerate mutase	No
SSA_0859	7.75	62.77	Triosephosphate isomerase	No
SSA_0813	9.7	11.04	Thioredoxin reductase	No
SSA_0772	9.91	75.4	PTS family porter, phosphocarrier protein HPR	No
SSA_0878	11.11	16.27	DNA gyrase subunit B	No
SSA_1896	11.8	32.13	Translation initiation factor IF-2	No
SSA_1043	12.99	5.47	Homoserine dehydrogenase	No
SSA_0636	14.5	6.01	N-(5′-phosphoribosyl)anthranilate isomerase	No
SSA_0437	14.64	23.25	30S ribosomal protein S6	No
SSA_0522	48.94	10.91	Putative ethanolamine utilization protein EutM1	No

*Score provided by Progenesis software. By default, a score >5 indicates a statistically significant finding according to the following parameters for protein identification: allowed missed cleavages by trypsin set to 1; fixed modifications by carbamidomethyl (C); variable oxidation (M) was allowed; at least 2 peptides per protein; 4 fragments per peptide; 10 fragments per protein.

†As predicted by LipoP 1.0 software.

Reduced-abundance proteins were involved in several functional categories, as described by the KEGG database. Two of these proteins (SSA_0291 and SSA_1938) are predicted to be involved in fatty acid biosynthesis, where SSA_0291, a short-chain dehydrogenase, was predicted to be a SPase substrate.

Many proteins putatively involved in peptidoglycan biosynthesis were reduced in abundance. Glutamine and glutamate are major contributors to peptidoglycan biosynthesis and proteins involved in their metabolism impact on cell wall formation, as described by the KEGG database. The reduced abundance of protein SSA_0371, involved in glutamate biosynthesis, implied a deficiency of glutamine. Another related protein, SSA_0570, is needed for the addition of glutamine to the elongating peptides during translation. Moreover, the enzymes (SSA_2107 and SSA_2183) involved in the synthesis of peptidoglycan (glucosamine 6-phosphate) were also reduced. Although none of the above-mentioned proteins involved in peptidoglycan biosynthesis was a predicted SPase substrate, their reduced abundance in Δ*SSA_0351* indirectly implies an SPase contribution to cell wall biosynthesis.

Finally, two enzymes needed for gluconeogenesis were reduced in Δ*SSA_0351* – SSA_1012 and SSA_1528 – emphasizing the need for glycolysis and energy production. In contrast, 10 enzymes needed for energy production through glycolysis and lactate fermentation were increased significantly. Interestingly, lactate dehydrogenase (SSA_1221), which is responsible for cofactor NAD+synthesis at a post-glycolysis step, is a predicted SPase substrate.

Other up-regulated proteins were involved in oligopeptide transport and chaperone activity. Two components of oligopeptide ABC transporter (SSA_1950 and SSA_1948) were up-regulated in Δ*SSA_0351*. Both proteins were predicted to have transmembrane domains and their aberrant abundance in the cytoplasm would suggest a shortage of amino acids in Δ*SSA_0351*.

Up-regulation of chaperones (SSA_1998, SSA_0226, SSA_2007, SSA_0141) is consistent with a stressful impact of misfolded proteins on the proteostasis in Δ*SSA_0351*.

To conclude, proteomic findings suggested a role for SPase in cell wall integrity through the involvement of SPase targets in fatty acid and peptidoglycan biosynthesis.

### Comparative analysis of biofilm formation by SPase-target mutants

**Fig. 6.**
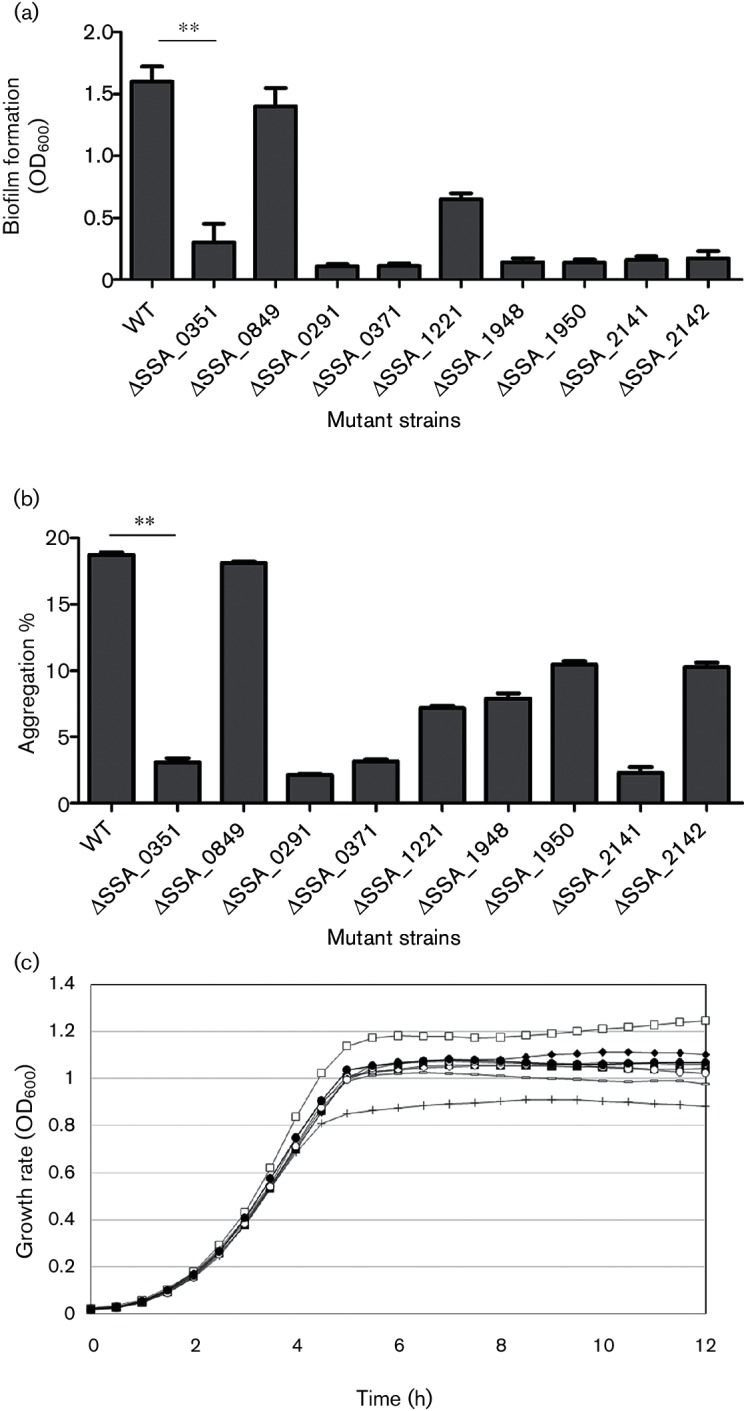
Evaluation of the biofilm formation potential of SPase SSA_0351 targets. (a) Potential targets of SPase *SSA_0351* were shown to be involved in biofilm formation, as shown by the biofilm assays of their respective mutants. Bacterial samples of eight replicates each were cultured anaerobically for 24 h in BM with 1 % sucrose. After crystal violet staining, biofilm formation was quantified at OD_600_ and the results were compared using ANOVA and the multiple comparison method (Dunnett's test). Statistically significant results had a cutoff *P*-value <0.05. **indicates significance with *P*-value <0.01. (b) Evaluation of the auto-aggregation ability of potential targets of *SSA_0351*-encoded SPase. The auto-aggregation ability of *S. sanguinis* mutants with respect to the wild-type was measured using the auto-aggregation assay, where the absorbance of each bacterial culture was measured at the time points 0 and 8 h. Auto-aggregation ability was expressed as a percentage. (c) Growth curves of mutants of differentially regulated genes/proteins in Δ*SSA_0351*. The growth rates of the wild-type strain and mutants were compared at different time intervals (hours). The growth curve assay was performed in triplicate and the average of the three experiments is shown here. Legends: empty square, WT; filled square, *ΔSSA_1948;* empty circle, *ΔSSA_1221*; filled circle, *ΔSSA_0291*; empty rhombus, *ΔSSA_1950*; filled rhombus, *ΔSSA_0371*; plus sign, *ΔSSA_2141*; dash, *ΔSSA_2141*.

Collectively, all genes in Δ*SSA_0351* that displayed reduced mRNA expression, as determined by RNA-seq, or protein production, as quantified by mass spectrometry, were further investigated for their contribution to biofilm formation. Mutants of these respective genes were tested for biofilm formation using the biofilm assay (Fig. 6a). Most mutants whose deleted genes are predicted to be involved in peptidoglycan formation (Δ*SSA*_*2141*, Δ*SSA*_*2142* and Δ*SSA*_*0371*), fatty acid biosynthesis (Δ*SSA*_0291) and ABC transport of amino acids (Δ*SSA*_*1950* and Δ*SSA*_*1948*) showed reduced biofilm formation, as determined by the biofilm assay (Fig. 6a), connecting the absence of SPase in Δ*SSA*_*0351* with reduced biofilm formation (Fig. 2a, b).

Moreover, the same mutants were tested for their auto-aggregation ability using the auto-aggregation assay, where a decrease in the turbidity of a standing culture, calculated as an increase in aggregation percentage, corresponded to an increase in bacterial aggregate formation, the first step in the biofilm formation process. Wild-type cells in long chains aggregated at an aggregation percentage of 18.6 %, which was comparable to that of Δ*SSA* _*0849* (18.3 %), while all of the other mutants showed significantly (*P*-value<0.001) lower aggregation percentages, reflecting a defect in aggregation ability (Fig. 6b) that may be attributed to many factors, such as short chains, as suggested by our previous study [30]. Finally, the growth rates of all the SPase-target mutants were compared to that of the wild-type to rule out a causal relationship between the growth rate reduction of any mutant and reduced biofilm formation. All of the mutants exhibited comparable growth rates in comparison to the wild-type (Fig. 6c).

## Discussion

Oral biofilm formation in streptococci has been shown to be involved in a variety of microbial infections in the human body, through recruiting diverse bacterial species to the site of infection and displaying an effective defence system against host immune defences [1]. *S. sanguinis* has been shown to be involved in biofilm formation and we hypothesized that SPases are a main contributor to this biological phenomenon. Investigating the role of SPase in biofilm formation may uncover potential drug targets against diverse bacterial infections that involve biofilm formation in the oral cavity.

The functional analysis of SPases in Gram-positive bacteria is limited to a few model bacterial species, such as *Bacillus subtilis* [20], *Bacillus amyloliquefaciens* [31], *Streptomyces lividans* [32], *Streptococcus pneumoniae* [33] and *Staphylococcus aureus* [34]. We used data mining to extract functional clues about SPases in *S. sanguinis*. In concurrence with most Gram-positive bacteria that contain multiple SPases – reaching up to seven in *Bacillus cereus*, six in *Bacillus anthracis* and five in *B. subtilis* [20, 21, 35, 36] – *S. sanguinis* harbours two SPases, encoded by *SSA_0351* and *SSA_0849*. Although these SPases share 31 % protein sequence identity [25], substrate overlap between both SPases and its impact on biofilm formation remained a challenging question. Multiple SPases within many Gram-positive bacteria display substrate overlap with different processing efficiencies, such as the six SPases of *B. subtilis* with respect to the processing of a β-lactamase precursor [20], and *L. monocytogenes* SipX and SipZ with respect to the processing of phospholipase C [37]. We hypothesized that *S. sanguinis* SPases should demonstrate substrate overlap based on the fact that neither of them is essential (either of them can be knocked out) although, based on LipoP software prediction, they process the essential proteins SSA_1604 (preprotein translocase subunit SecG) and SSA_0941 (phosphate ABC transporter substrate-binding protein). Using our library of *S. sanguinis* knockouts, we were prompted to examine the impact of *ΔSSA_0351* and Δ*SSA_0849* on biofilm formation. We identified *SSA_0351*-encoded SPase as the main contributor to biofilm formation in *S. sanguinis,* which agrees with the findings in other Gram-positive bacterial models in which SPases were shown to regulate biofilm formation: SipW in *B. subtilis* [38] and LepB2 in *Actinomyces oris* [39].

To date no experimental data link *S. sanguinis* SPases to their predicted substrates. Phenotypic observations of Δ*SSA_0351* biofilms provided hints about potential SPase substrates. SEM images showed the absence of lengthy chains and aberrant cell morphologies in Δ*SSA_0351*, while CLSM revealed the inability of Δ*SSA_0351* cells to aggregate and initiate biofilm formation. Our primary suggestion regarding the observation of aberrant cell walls was that genes involved in cell wall biosynthesis, including peptidoglycan and fatty acid biosynthesis, may be potential targets of *SSA_0351*-encoded SPase, as previous findings have linked the synthesis of peptidoglycan or other cell wall components to changes in cell morphology [40]. Moreover, previous work in our laboratory linked short chain length in *S. sanguinis*, which was observed in SEM images of Δ*SSA_0351*, with inefficient aggregation [30]. In addition, the *S. aureus* SPase, SpsB, was shown to process a quorum sensing protein, AgrD, *in vitro*, which was needed for biofilm formation and virulence [41]. Based on our transcriptomic and proteomic findings, we did not identify any significant changes of quorum-sensing components in Δ*SSA_0351*. However, the overexpression of oligopeptide transporters may be harnessed for the uptake of quorum sensing elements, which may be linked to the severe reduction in aggregation potential of Δ*SSA_0351*, as shown by auto-aggregation assay. Finally, a proteomic approach aimed at isolating membrane proteins in *S. sanguinis* is under development. It may be insightful to quantify differential membrane protein expression in Δ*SSA_0351*, complementing our findings with cytosolic protein expression in the same mutant.

Surprisingly, Terra *et al*. [29] showed that although the *Bacillus subtilis* SPase, SipW, was required for biofilm formation, its signal peptidase activity was not required for solid-surface biofilms. Although we do not substantiate that *SSA_0351*-encoded SPase impacts on biofilm formation through its signal peptidase activity, we believe that this model may be the most justifiable in light of our current findings. Although both SPases, SipW and SSA_0351, affect biofilm formation, the percentage identity of their protein sequence alignment was the lowest among the proteins compared (Fig. 1b). In this study, we conducted the transcriptomic and proteomic assays using planktonic cells because ΔSSA_0351 did not form biofilm and therefore could not be compared in any aspect to the wild-type cells in a biofilm.

We identified two potential SPase targets, SSA_0291 and SSA_1221, whose cytoplasmic protein levels were impacted on by the absence of SPase SSA_0351. The SSA_0291 protein level was decreased (to 0.31-fold of the wild-type level). In contrast, the protein level of another putative SPase substrate, SSA_1221, was elevated (~fivefold of the wild-type level). The different protein levels may be from different protein degradation mechanisms in ΔSSA_0351. To understand this protein level difference, we investigated further protein folding and degradation machinery in the ΔSSA_0351 proteome. We found that the SPase proteome showed a significant up-regulation of the trigger factor (SSA_1998, 1.14-fold) and DnaK (SSA_2007, 2.06-fold). In *E. coli*, DnaK and the trigger factor share the task of folding nascent polypeptides and prevent the entry of these emerging polypeptides into ‘kinetic traps’ during their folding intermediates. It was shown previously that the DnaK chaperone system favours substrates with an isoelectric point range between 5 and 7 [42]. We found that the PI values for SSA_0291 and SSA_1221 proteins were 9.3 and 5.15, respectively. The DnaK chaperone system might therefore have been biased towards interacting with SSA_1221 and protecting it from degradation. Thus, we predict that SSA_0291 is degraded and so reduced in abundance in the ΔSSA_0351 mutant, whereas SSA_1221 is protected from degradation and so accumulates in the cytoplasm rather than being secreted. If secretion is important for the function of SSA_1221 in relation to biofilm formation, this would explain why both increased cytoplasmic abundance of SSA_1221 in the ΔSSA_0351 mutant and reduced abundance due to deletion of the SSA_1221 gene results in aberrant biofilm formation. Additional experiments will be required to assess this model.

In addition, lactate dehydrogenase (Ldh; SSA_1221) is responsible for cofactor NAD+synthesis at a post-glycolysis step. It is possible that the up-regulation of the (NAPD+)-dependent enzyme GapN (SSA_0774) at the expense of the (NAD+)-dependent enzyme GapA (SSA_2108), although both enzymes react with the same substrate (glyceraldehyde 3-phosphate), may be attributed to a decrease in the NAD+ concentration, related to a nonfunctional Ldh. Moreover, the KEGG database shows that many enzymes are (NAD+)-dependent, such as SSA_1047 (MurB involved in peptidoglycan biosynthesis), SSA_2168 (GpsA involved in glycerophospholipid biosynthesis) and SSA_1938 (FabK involved in fatty acid biosynthesis). We suggest that a nonfunctional lactate dehydrogenase may be impacting on the biosynthesis of cell wall components through a decrease in the activity of NAD+-dependent enzymes.

We further tested the auto-aggregation potential of the SPase substrate mutants Δ*SSA_0291* and Δ*SSA_1221,* as this reflects the bacteria–bacteria binding potential that is needed for biofilm formation, and the two knockouts showed a statistically significant reduction (*P*-value<0.0001) in aggregation percentage, as compared to wild-type. Moreover, phenotypic analysis of biofilm formation through microtitre plate assay showed a total absence of biofilms in Δ*SSA*_0291 and Δ*SSA*_1221. Further phenotypic investigation through CLSM imaging will provide a better understanding of biofilm formation in these mutants.

In addition, many transcriptionally down-regulated genes (*SSA_2141* and *SSA_2142*), which may be attributed to a deficiency of glutamine, the fundamental precursor for arginine biosynthesis in *S. sanguinis* SK36, and down-regulated proteins (SSA_0371), showed reduced biofilm formation (Fig. 6a) as well as aggregation percentages (Fig. 6b). However, the knockout mutants of these genes displayed comparable growth rates with respect to the wild-type (Fig. 6c). We suggested that although these gene products were not predicted SPase substrates, an indirect link between SPase and these biofilm regulators may be inferred. Finally, many down-regulated proteins (SSA_1938, SSA_0570, SSA_2107 and SSA_2183) could not be knocked out to have their mutants tested for biofilm formation, due to their essentiality.

In conclusion, this study demonstrated that the SPase mutant Δ*SSA_0351* (but not Δ*SSA_0849*) caused a decrease in biofilm formation compared to the wild-type, as shown via the biofilm assay and CLSM imaging. SEM imaging, along with transcriptomics and proteomics experiments, strongly suggested a deformation of the cell wall that could be attributed to destabilized peptidoglycan, dysfunctional transporters, and reduced glycerolipid and glycerophospholipid biosynthesis. Future studies may delve deeper into the intricacies of biofilm formation by defining the exact substrates of each SPase through signal peptidase activity and progress our understanding of the regulatory pathways that control the function and structure of the biofilm matrix.

## Supplementary Data

177 Supplementary File 1
