# Supplementary material for: Involvement of signal peptidase I in Streptococcus sanguinis biofilm formation
Source: Microbiology (Reading). 2017 Sep 4;163(9):1306–18. doi: 10.1099/mic.0.000516 (PMC5817204; doi:10.1099/mic.0.000516)
Supplement: Supplementary File 1 [file mic-163-1306-s001.pdf]

S1 table. Description of potential SPase substrates as predicted by LipoP software**.**

Gene symbol	Gene description
SSA_0012	Beta-lactamase class A, putative
SSA_0015	Membrane ATPase FtsH, degrades sigma32 (integral membrane cell-division Zn metallo-peptidase), putative
SSA_0017	Cell shape determining protein MreC, putative
SSA_0019	Secreted antigen GbpB/SagA; peptidoglycan hydrolase; PcsB protein precursor, putative
SSA_0021	hypothetical protein SSA_0021
SSA_0036	Secreted protein, possible function in cell-wall metabolism (amidase), putative
SSA_0094	Cell wall metabolism, LysM type protein, putative
SSA_0140	Copper-translocating P-type ATPase, putative
SSA_0146	DNA repair ATPase, putative
SSA_0157	hypothetical protein SSA_0157
SSA_0165	hypothetical protein SSA_0165
SSA_0167	Hypothetical protein (Asparagine/proline-rich)
SSA_0173	23S rRNA m1G745 methyltransferase, putative
SSA_0175	Penicillin-binding protein 1B, putative
SSA_0181	Glycosyltransferase (vectorial glycosyl polymerization (VGP) family), putative
SSA_0186	Competence protein ComYC, putative
SSA_0187	Competence protein ComYD, putative
SSA_0189	Competence protein ComGF, putative
SSA_0210	hypothetical protein SSA_0210
SSA_0215	Periplasmic sugar-binding protein (ribose porter), putative
SSA_0218	Sugar-binding periplasmic protein, putative
SSA_0227	Collagen-binding surface protein, putative
SSA_0243	2',3'-cyclic nucleotide 2'-phosphodiesterase/3'-nucleotidase bifunctional periplasmic precursor protein
SSA_0257	N-acetylmuramidase/lysin, putative
SSA_0273	hypothetical protein SSA_0273
SSA_0291	short chain dehydrogenase

SSA_0301	hypothetical protein SSA_0301
SSA_0303	surface protein C
SSA_0304	Bacterial cell wall degradation (CHAP/LysM domains), putative
SSA_0351	Signal peptidase I, putative
SSA_0396	hypothetical protein SSA_0396
SSA_0398	hypothetical protein SSA_0398
SSA_0400	hypothetical protein SSA_0400
SSA_0424	Exopolysaccharide biosynthesis protein, putative
SSA_0453	Type II secretory pathway, pullulanase PulA glycosidase, putative
SSA_0477	Cobalamin biosynthesis protein CbiM (ABC-type cobalt transporter), putative
SSA_0478	Cobalt transport protein cbiN, putative
SSA_0498	ABC-type dipeptide/oligopeptide/nickel transport systems, permease components, putative
SSA_0500	Peptide ABC transporter, permease protein, putative
SSA_0521	Ethanolamine utilization protein EutL, putative
SSA_0565	hypothetical protein SSA_0565
SSA_0607	ABC transporter, permease component, putative
SSA_0610	LemA-like protein, putative
SSA_0613	Glucosyltransferase, putative
SSA_0684	Fibril-like structure subunit FibA, putative
SSA_0689	Penicillin-binding protein 2B, putative
SSA_0723	hypothetical protein SSA_0723
SSA_0726	FmtA-like protein, putative
SSA_0747	DD-carboxypeptidase, putative
SSA_0793	DNA-entry nuclease, putative
SSA_0801	Mur ligase family protein, putative
SSA_0803	hypothetical protein SSA_0803
SSA_0805	Collagen-binding surface protein, putative
SSA_0871	Cell division protein FtsX, putative
SSA_0881	Lipoprotein, putative
SSA_0897	Two component system histidine kinase, putative
SSA_0904	CshA-like fibrillar surface protein A

SSA_0905	CshA-like fibrillar surface protein B
SSA_0906	CshA-like fibrillar surface protein C
SSA_0908	ABC-type uncharacterized transport system, periplasmic component, putative
SSA_0947	hypothetical protein SSA_0947
SSA_0956	surface protein D
SSA_0963	Peptidoglycan N-acetylglucosamine deacetylase A, putative
SSA_0967	hypothetical protein SSA_0967
SSA_0970	hypothetical protein SSA_0970
SSA_0991	Deoxyribonuclease, putative
SSA_1018	Zinc metalloprotease zmpC precursor, putative
SSA_1019	Collagen-binding surface protein, putative
SSA_1023	Von Willebrand factor-binding protein precursor, putative
SSA_1042	Xylanase/chitin deacetylase, putative
SSA_1051	Spermidine/putrescine ABC transporter, spermidine/putrescine-binding protein, putative
SSA_1052	hypothetical protein SSA_1052
SSA_1063	Peptidoglycan-binding domain-containing protein, putative
SSA_1064	hypothetical protein SSA_1064
SSA_1065	Beta-hexosamidase A, putative
SSA_1095	Peptidoglycan hydrolase, putative
SSA_1106	IgA-specific metalloendopeptidase
SSA_1112	Cell wall surface anchor family protein, putative
SSA_1118	Peptide methionine sulfoxide reductase, putative
SSA_1130	Iron-dependent peroxidase, putative
SSA_1132	TatC, sec-independent protein translocase, putative
SSA_1148	Beta-glucosides PTS, EIIC, putative
SSA_1158	hypothetical protein SSA_1158
SSA_1161	hypothetical protein SSA_1161
SSA_1219	Sortase, putative
SSA_1221	L-lactate dehydrogenase
SSA_1234	5'-nucleotidase, putative
SSA_1274	hypothetical protein SSA_1274
SSA_1301	Conserved uncharacterized protein, possible surface protein
SSA_1339	Pneumococcal histidine triad protein D

	precursor, putative
SSA_1359	Arginine/histidine ABC transporter, permease component, putative
SSA_1363	FmtA-like protein, putative
SSA_1365	FmtA-like protein, putative
SSA_1368	hypothetical protein SSA_1368
SSA_1369	FmtA-like protein, putative
SSA_1371	FmtA-like protein, putative
SSA_1372	hypothetical protein SSA_1372
SSA_1390	hypothetical protein SSA_1390
SSA_1391	hypothetical protein SSA_1391
SSA_1408	hypothetical protein SSA_1408
SSA_1415	Oxidoreductase, putative
SSA_1434	Conserved uncharacterized Firmicutes protein
SSA_1481	FmtA-like protein, putative
SSA_1489	hypothetical protein SSA_1489
SSA_1525	Lyzozyme M1 (1,4-beta-N-acetylmuramidase), putative
SSA_1532	Membrane-fusion protein / periplasmic component of efflux system, putative
SSA_1544	Conserved uncharacterized protein
SSA_1567	Polar amino acid ABC transporter, amino acid-binding protein, putative
SSA_1588	Conserved ABC-type antimicrobial permease-like protein, putative
SSA_1591	Dipeptidase, putative
SSA_1593	Dipeptidase, putative
SSA_1594	Metalloendopeptidase, putative
SSA_1596	hypothetical protein SSA_1596
SSA_1597	hypothetical protein SSA_1597
SSA_1598	hypothetical protein SSA_1598
SSA_1599	hypothetical protein SSA_1599
SSA_1626	DNA translocase ftsK, putative
SSA_1631	Sortase-like protein, putative
SSA_1632	Surface protein, putative
SSA_1633	FimA fimbrial subunit-like protein, putative
SSA_1634	Heme utilization/adhesion exoprotein, putative
SSA_1635	hypothetical protein SSA_1635
SSA_1649	hypothetical protein SSA_1649
SSA_1650	3-ketoacyl-(acyl-carrier-protein) reductase
SSA_1653	hypothetical protein SSA_1653
SSA_1663	Collagen-binding protein A

SSA_1671	hypothetical protein SSA_1671
SSA_1673	hypothetical protein SSA_1673
SSA_1680	ABC-type bacitracin resistance protein A, permease component, putative
SSA_1692	Phospho-B-galactosidase LacG, putative
SSA_1744	Iron compound ABC transporter, permease protein, putative
SSA_1750	Extracellular nuclease, putative
SSA_1793	Histidine kinase (sensor protein), putative
SSA_1871	Penicillin-binding protein 2X, putative
SSA_1882	Subtilisin-like serine proteases, putative
SSA_1909	Transcriptional attenuator LytR, putative
SSA_1951	Penicillin-binding protein 3, putative
SSA_1960	hypothetical protein SSA_1960
SSA_1961	Amino acid ABC transporter, amino acid-binding protein/permease protein, putative
SSA_1984	Cell surface SD repeat antigen precursor, putative
SSA_1985	hypothetical protein SSA_1985
SSA_1991	Pneumococcal histidine triad protein A, putative
SSA_2004	Zinc metalloprotease zmpB precursor, putative
SSA_2014	D-alanyl-D-alanine carboxypeptidase, putative
SSA_2020	hypothetical protein SSA_2020
SSA_2023	Fructan beta-fructosidase precursor, putative
SSA_2056	Cinnamoyl ester hydrolase, putative
SSA_2060	Arabinose efflux permease, putative
SSA_2074	preprotein translocase subunit YajC
SSA_2101	Amino acid ABC transporter, periplasmic amino acid-binding protein, putative
SSA_2103	hypothetical protein SSA_2103
SSA_2121	Cell wall surface anchor family protein, putative
SSA_2169	Glucose-1-phosphate uridylyltransferase, putative
SSA_2248	hypothetical protein SSA_2248
SSA_2250	ABC-type antimicrobial peptide transporter, permease component, putative
SSA_2264	hypothetical protein SSA_2264
SSA_2269	hypothetical protein SSA_2269
SSA_2281	hypothetical protein SSA_2281
SSA_2282	Phage infection protein, putative

SSA_2301	S-layer protein/ peptidoglycan endo-beta-N-acetylglucosaminidase, putative
SSA_2307	hypothetical protein SSA_2307
SSA_2313	hypothetical protein SSA_2313
SSA_2320	hypothetical protein SSA_2320
SSA_2321	Cation (Co/Zn/Cd) efflux protein, putative
SSA_2338	Conserved uncharacterized protein
SSA_2340	hypothetical protein SSA_2340
SSA_2364	Immunodominant staphylococcal antigen A precursor, putative
SSA_2381	DegP protein, putative

S2 table. Microtitter plate readings for wild type and mutant strains before (upper table) and after (lower table) crystal violet staining.

	**WT**	**Δ0849**	**Δ0351**	**Δ0291**	**Δ0371**	**Δ1948**	**Δ1950**	**Δ2141**	**Δ2142**
read # 1	0.846	0.82	0.886	0.915	0.733	0.885	0.915	0.81	0.831
read # 2	0.909	0.889	0.888	0.867	0.761	0.927	0.962	0.799	0.849
read # 3	0.905	0.88	0.937	0.963	0.83	0.88	1.061	0.745	0.904
read # 4	0.965	0.909	0.785	0.96	0.772	0.872	1	0.843	0.906
read # 5	0.911	0.902	0.956	0.904	0.839	0.87	1.071	0.741	0.86
read # 6	0.896	0.852	0.955	0.988	0.919	0.966	1.079	0.973	0.96
read # 7	0.879	0.897	0.963	0.904	0.872	0.929	1.093	0.819	0.902
read # 8	0.84	0.852	0.919	0.859	0.763	0.871	0.991	0.827	0.923
average	0.893875	0.875125	0.911125	0.92	0.811125	0.9	1.0215	0.819625	0.891875
P-value		0.31073	0.504805	0.247684	0.00793	0.751848	0.00029	0.023165	0.924001

	**WT**	**Δ0849**	**Δ0351**	**Δ0291**	**Δ0371**	**Δ1948**	**Δ1950**	**Δ2141**	**Δ2142**
read # 1	1.028	2.168	0.132	0.107	0.105	0.126	0.11	0.109	0.166
read # 2	1.46	1.99	0.091	0.101	0.103	0.105	0.095	0.101	0.095
read # 3	1.55	1.982	0.097	0.107	0.118	0.107	0.102	0.107	0.108
read # 4	1.303	2.169	0.104	0.103	0.118	0.107	0.113	0.083	0.113
read # 5	1.776	2.108	0.106	0.098	0.105	0.115	0.089	0.098	0.111
read # 6	1.755	1.821	0.105	0.093	0.104	0.103	0.102	0.096	0.111
read # 7	1.463	1.85	0.098	0.095	0.103	0.113	0.09	0.109	0.102
read # 8	1.327	1.622	0.106	0.108	0.141	0.124	0.136	0.119	0.123
average	1.45775	1.96375	0.104875	0.1015	0.112125	0.1125	0.104625	0.10275	0.116125
P-value		0.000418	3.21E-10	3.07E-10	3.46E-10	3.43E-10	3.23E-10	3.13E-10	3.72E-10
